# Combating Inflammation and Promoting Anabolism in Osteoarthritic Cartilage Defect With an MMP13‐Sensing Dual‐Drug Scaffold

**DOI:** 10.1002/advs.202519950

**Published:** 2026-07-13

**Authors:** Zhen Zhang, Bangheng Liu, Yulei Mu, Huiqun Zhou, Liang Ma, Chenjie Xu, Dong‐An Wang

**Affiliations:** ^1^ Department of Biomedical Engineering City University of Hong Kong Kowloon Hong Kong SAR China; ^2^ Department of Biomedical Engineering Chinese University of Hong Kong Sha Tin, New Territories Hong Kong SAR China; ^3^ Center for Neuromusculoskeletal Restorative Medicine InnoHK HKSTP Sha Tin Hong Kong SAR China

**Keywords:** anti‐inflammatory, cartilage defect, MMP13‐sensing, osteoarthritic, promoting anabolism

## Abstract

Osteoarthritis (OA) is a widespread degenerative joint condition marked by progressive cartilage breakdown, and a chronic inflammatory microenvironment, where conventional therapies largely fail to halt disease progression. To address this unmet need, an intra‐articularly implantable, disease‐responsive scaffold was developed for combinatorial treatment to simultaneously combat inflammation and promote anabolism. The system is based on an MMP13‐sensing peptide‐modified type II collagen scaffold engineered for controlled release of celecoxib (CXB), an anti‐inflammatory agent, and fibroblast growth factor‐18 (FGF‐18), a pro‐anabolic growth factor. Comprehensive physicochemical characterization confirmed the scaffold's porous structure, successful conjugation of the responsive peptide, and MMP13‐dependent drug release. In vitro studies demonstrated excellent biocompatibility, potent anti‐inflammatory effects, and enhanced chondrogenic matrix production under IL‐1β stimulation. When evaluated the rat OA cartilage defect model, the dual‐drug scaffold significantly suppressed inflammation and subchondral bone damage, while promoting early matrix anabolism, and outperforming the control group. This MMP13‐sensing scaffold represents a precision medicine strategy for OA therapy, enabling intelligent, microenvironment‐driven drug delivery to disrupt the degenerative cycle and facilitate synergistic early‐stage anti‐inflammatory and anabolic effects.

## Graphical abstract

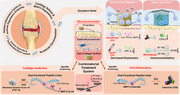

## 1 Introduction

Osteoarthritis (OA) is a globally prevalent disease, affecting over 500 million people. Consequently, it remains a leading cause of disability, especially among the aging population [1]. The disease is characterized by progressive degradation of articular cartilage, synovial inflammation, and alterations in subchondral bone architecture. OA pathogenesis involves a complex interplay of mechanical, cellular, and inflammatory processes that drive progressive joint deterioration. Inflammatory mediators like interleukin‐1β (IL‐1β) and tumor necrosis factor‐α (TNF‐α) exacerbate this process, stimulating the production of matrix‐degrading enzymes such as matrix metalloproteinases (MMPs), particularly MMP13, a master regulator of type II collagen destruction [1, 2]. This leads to the breakdown of key extracellular matrix (ECM) components such as glycosaminoglycans (GAGs) and type II collagen, ultimately compromising joint integrity and function [3]. Current OA management strategies include nonsteroidal anti‐inflammatory drugs (NSAIDs), intra‐articular corticosteroids, hyaluronic acid injections, and arthroplasty, which are limited in their ability to modify disease progression. These approaches primarily offer symptomatic relief and are constrained by systemic side effects, short intra‐articular residence half‐lives, lack of targeted delivery, and inability to simultaneously address both inflammation and tissue repair [4]. There is therefore an urgent need for targeted, disease‐modifying therapies that can effectively disrupt the degenerative OA microenvironment and promote functional cartilage restoration.

**SCHEME 1:**
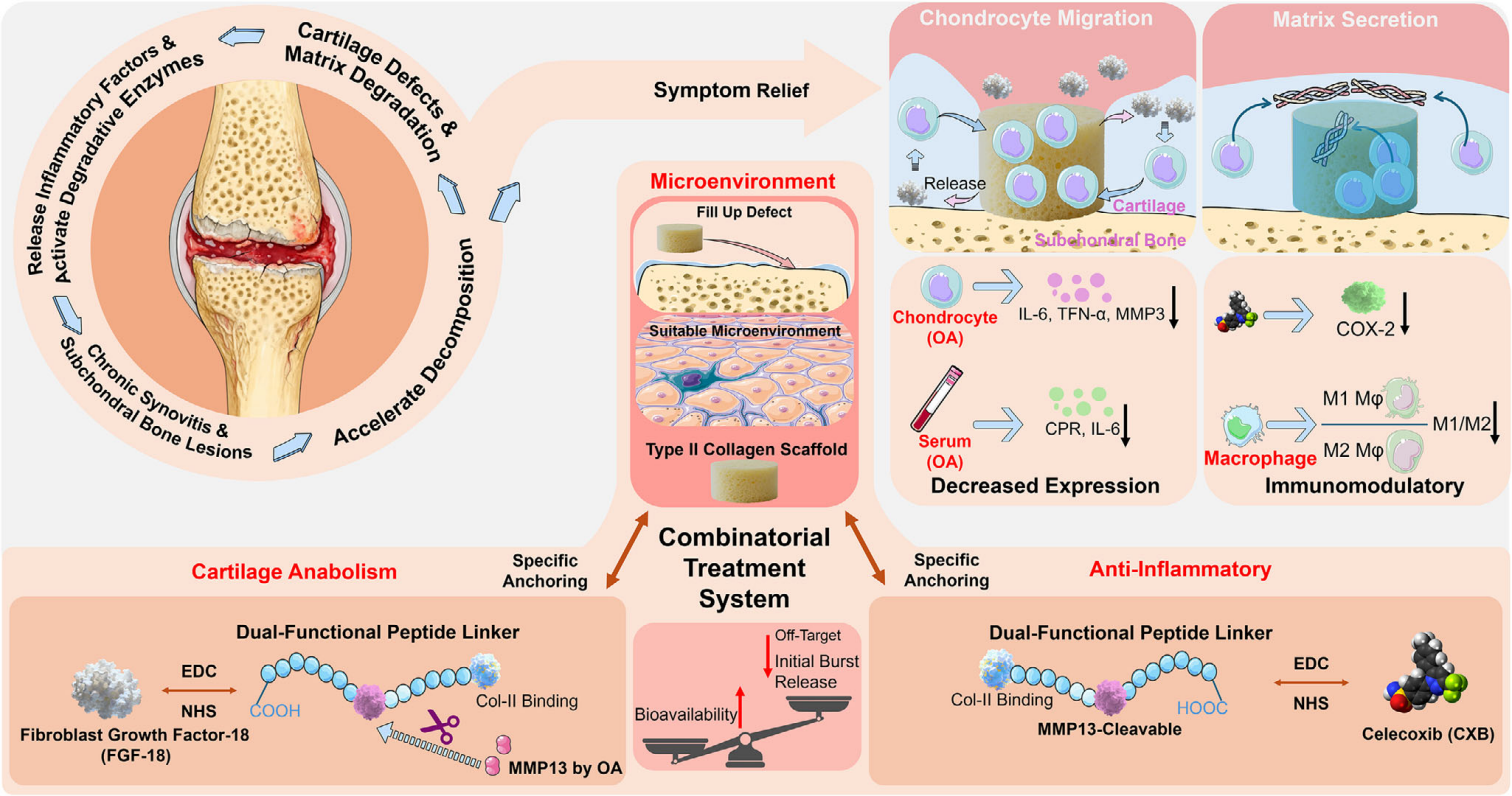
Osteoarthritic cartilage defect vicious cycle and its pathological basis and design concept of the MMP13‐sensing dual‐drug scaffold combating inflammation and promoting anabolism in osteoarthritic cartilage defect.

The challenge of achieving functional repair is further exacerbated by a vicious cycle (Scheme 1) that perpetuates joint degeneration. Initial cartilage loss exposes ECM components in OA defects, intensifying synovial inflammation and MMP13 secretion [3]. This heightened inflammatory and catabolic state accelerates further matrix destruction, deepening the defect and creating a self‐sustaining loop of degradation [5, 6, 7]. Therefore, a transformative therapeutic strategy must be multi‐faceted, capable of simultaneously quenching the inflammatory drive while physically bridging the defect with a scaffold that actively promotes repair. To break this cycle, a multifunctional scaffold was engineered based on type II collagen, the primary organic component of the native cartilage matrix, to enhance biocompatibility and chondrocyte interaction [8]. This biomimetic design is strategically intended to break the degenerative cycle through two critical mechanisms: first, by serving as a platform for the localized, on‐demand release of the anti‐inflammatory agent Celecoxib (CXB) to disrupt the cycle of inflammation [9]; and second, by acting as a biocompatible, 3D matrix that fills the defect, providing structural support and delivering fibroblast growth factor‐18 (FGF‐18) to create a potent pro‐anabolic microenvironment [10, 11]. This environment is designed to facilitate endogenous cell recruitment, proliferation, and differentiation, thereby actively initiating repair.

Central to this design is a dual‐functional peptide linker (WYRGRLGGPLGVRGG) that enables pathology‐responsive release of therapeutics. This linker features two distinct domains: an N‐terminal type II collagen‐specific binding peptide (WYRGRL) that firmly anchors the entire conjugate to the scaffold matrix [12], and a C‐terminal MMP13‐cleavable sequence (GPLG↓VR) [13, 14] that tethers the therapeutic agents: CXB, a selective COX‐2 inhibitor, and FGF‐18, a potent chondroinductive agent [10, 11]. This sophisticated architecture ensures that the drugs remain efficiently immobilized on the scaffold until encountering the pathologically elevated levels of MMP13 in the OA joint. Upon enzymatic cleavage, the therapeutics are released at the disease site, enabling a controlled therapeutic response. This design maximizes local drug bioavailability while minimizing off‐target effects.

It is hypothesized that this system will enable the synergistic action of CXB, which effectively inhibits inflammation and creates a favorable environment, and FGF‐18, which directly stimulates chondrocyte proliferation and matrix synthesis (Scheme 1). This combination aims to halt the degenerative cycle and promote early‐stage anabolism effectively. Herein, we present the development and evaluation of this MMP13‐sensing, type II collagen‐based dual‐drug scaffold. Its efficacy in combating inflammation, promoting anabolism, and facilitating early repair of osteochondral defects was systematically validated through comprehensive in vitro studies and in vivo assessments in a rat OA model over a 6‐week period. MMP13‐sensing type II collagen‐based scaffolds present a promising strategy for OA therapy by simultaneously combating inflammation and promoting anabolism in a single platform.

## 2 Results

### 2.1 Fabrication and Characterization of Scaffolds

**FIGURE 1:**
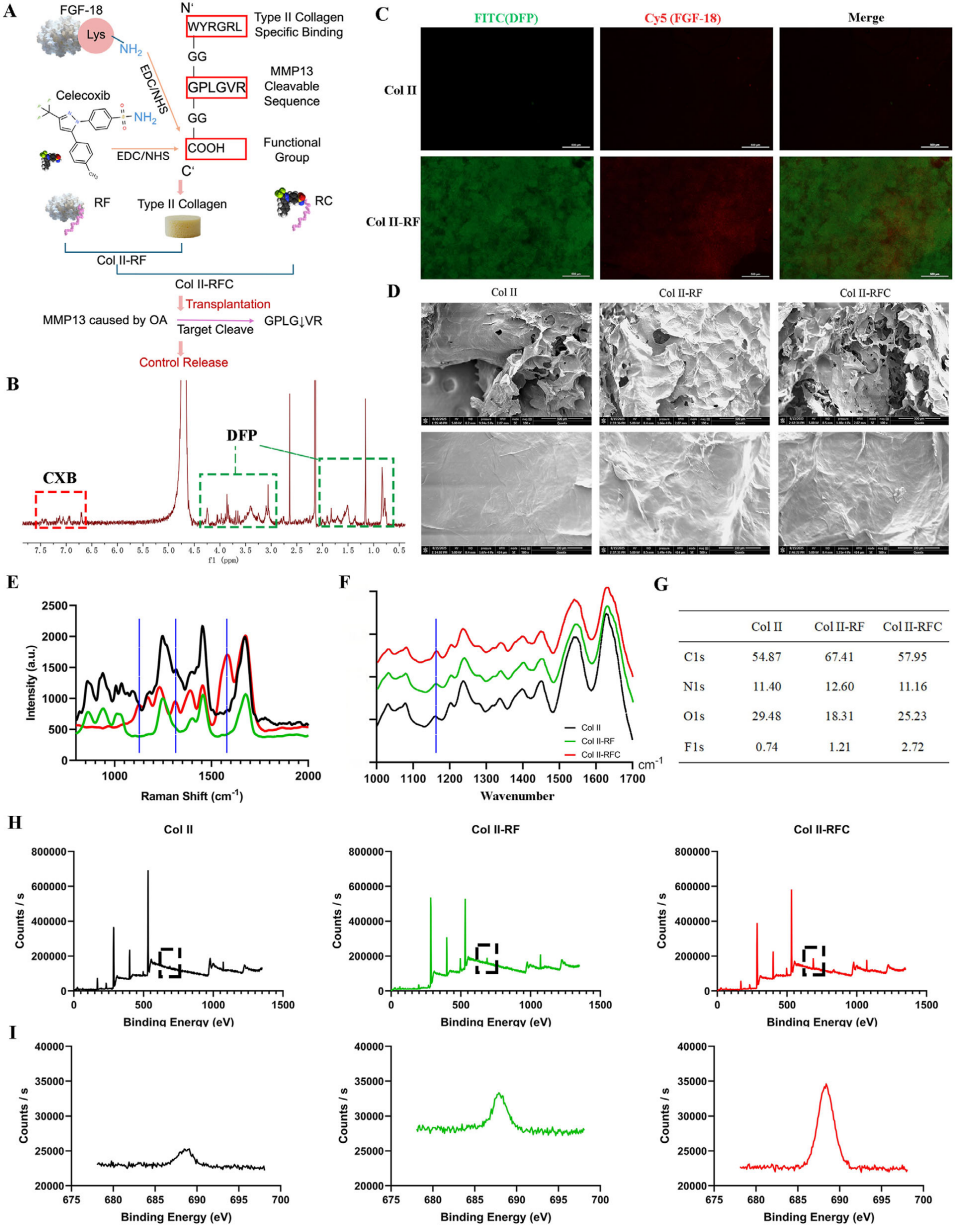
Fabrication and characterization of the MMP13‐sensing dual‐drug scaffold. (A) Schematic illustration of the scaffold design. (B) ^1^H NMR analysis of DFP functionalized with CXB (RC) in (CD_3_)_2_S═O. (C) Immunofluorescence co‐localization analysis demonstrates the successful binding of DFP (labeled with FITC, green) conjugated with FGF‐18 (labeled with Cy5, red) to the type II collagen scaffold (Col II‐RF). (D) Representative scanning electron microscopy (SEM) images showing the highly porous and interconnected microstructure of the Col II, Col II‐RF, and the final dual‐drug‐loaded scaffold (Col II‐RFC). (E) Raman spectra confirming the successful conjugation of the RF and RC to the type II collagen scaffold. (F) Fourier transform infrared (FTIR) spectra confirming the successful conjugation of the RF (DFP‐FGF‐18) and RC (peptide‐CXB) to the Col II scaffold. (G) X‐ray photoelectron spectroscopy (XPS) analysis clearly identifies the components of the scaffolds. (H) The spectra of the scaffolds and (I) the F1s spectra of the different scaffolds.

A novel MMP13‐sensing type II collagen scaffold was successfully engineered for the targeted co‐delivery of CXB and FGF‐18 to osteoarthritic cartilage defects. Figure 1A schematically illustrates the design of the system, which incorporates a dual‐functional peptide linker (DFP) for MMP13‐sensing drug release and binding with type II collagen.

The successful functionalization of the DFP with CXB (RC) was confirmed by ^1^H NMR spectroscopy (Figure 1B), which displays characteristic proton peaks corresponding to both the DFP and the CXB in (CD_3_)_2_S═O. The peaks between 0.5 and 2.0 ppm mainly correspond to the methyl and methylene protons of the aliphatic side chains in the DFP, while the peaks between 3.0 and 4.5 ppm mainly correspond to the α‐protons of the DFP. The peaks between 6.5 and 8.0 ppm originate from the aromatic ring protons in the CXB [15, 16]. Figure S1 shows the ^1^H NMR spectroscopy of CXB and DFP with FGF‐18 (RF). Furthermore, immunofluorescence co‐localization analysis using the type II collagen scaffold as a negative control demonstrated the efficient binding of the FITC‐labeled DFP (green) and the Cy5‐tagged FGF‐18 (red) to the type II collagen scaffold (Col II‐RF), validating the precise assembly of the targeting and delivery components (Figure 1C). This indicates that FGF‐18 was successfully grafted onto DFP, and the synthesized product RF still possesses the ability to bind the type II collagen scaffold. The specific binding ability of DFP (labeled with FITC, green) to type II collagen scaffolds is confirmed in Figure S2.

Morphological assessment via scanning electron microscopy (SEM) revealed that all scaffold groups, Col II, Col II‐RF, and the final dual‐drug‐loaded scaffold (Col II‐RFC), possessed a highly porous and interconnected 3D microstructure, which is critical for cell infiltration, nutrient diffusion, and tissue integration (Figure 1D). Compared with the Col II scaffold, the binding of RF and RC did not alter the scaffold morphology or pore size. The conjugation of the RF and RC to the Col II scaffold was verified through spectroscopic techniques. Raman spectroscopy (Figure 1E) and Fourier transform infrared (FTIR) spectroscopy (Figure 1F) both show the appearance of new vibrational bands characteristic of amide bonds and drug‐specific functional groups, indicating successful conjugation. In the Raman spectra, the Col II scaffold displays characteristic peaks of collagen: the band at 1250–1350 cm^−^^1^ corresponds to C–N stretching vibrations, reflecting the characteristics of peptide bonds; the band at 1640–1680 cm^−^^1^ corresponds to the C═O stretching vibration of the peptide bond (Amide I band), representing a key characteristic peak [17]. After RF binding, the Col II‐RF scaffold exhibits similar peak positions to those of the Col II scaffold, but with significantly reduced intensity. Following subsequent RC binding, the resulting Col II‐RFC scaffold shows peaks at 1550–1650 cm^−^^1^, corresponding to benzene rings, the peaks at 1100–1150 cm^−^^1^ and 1300–1350 cm^−^^1^ corresponding to symmetric and asymmetric stretching vibrations of the S═O, indicating the presence of CXB [18, 19]. In FTIR spectroscopy, comparing the Col II and Col II‐RF scaffolds reveals that the Col II‐RFC scaffold shows a peak at 1150–1160 cm^−^^1^, corresponding to symmetric stretching vibration of the S═O group [18, 19]. X‐ray photoelectron spectroscopy (XPS) analysis provides definitive evidence of the modified surface composition, with clear spectral signatures confirming the incorporation of the peptide‐drug conjugate onto the Col II scaffold. Elemental analysis indicates that the F1s signal, characteristic of CXB, is significantly increased in Col II‐RFC (Figure 1G). The spectra confirm that the overall composition is influenced by the presence of CXB in Col II‐RFC (Figure 1H,I). The FGF‐18 was indirectly assessed by measuring His‐tag content with a His Tag ELISA kit and celecoxib content was determined using a UV spectrophotometer. The results show the conjugation amount of RF on Col II‐RF is approximately 10.88 µg mg^−1^, and similarly, it is about 10.88 µg mg^−1^ on Col II‐RFC, and the conjugation amount of RC is 0.79 mg mg^−1^, with celecoxib accounting for 43.45 µg mg^−1^.

### 2.2 MMP13‐Sensing Drug Release and In Vitro Biocompatibility

**FIGURE 2:**
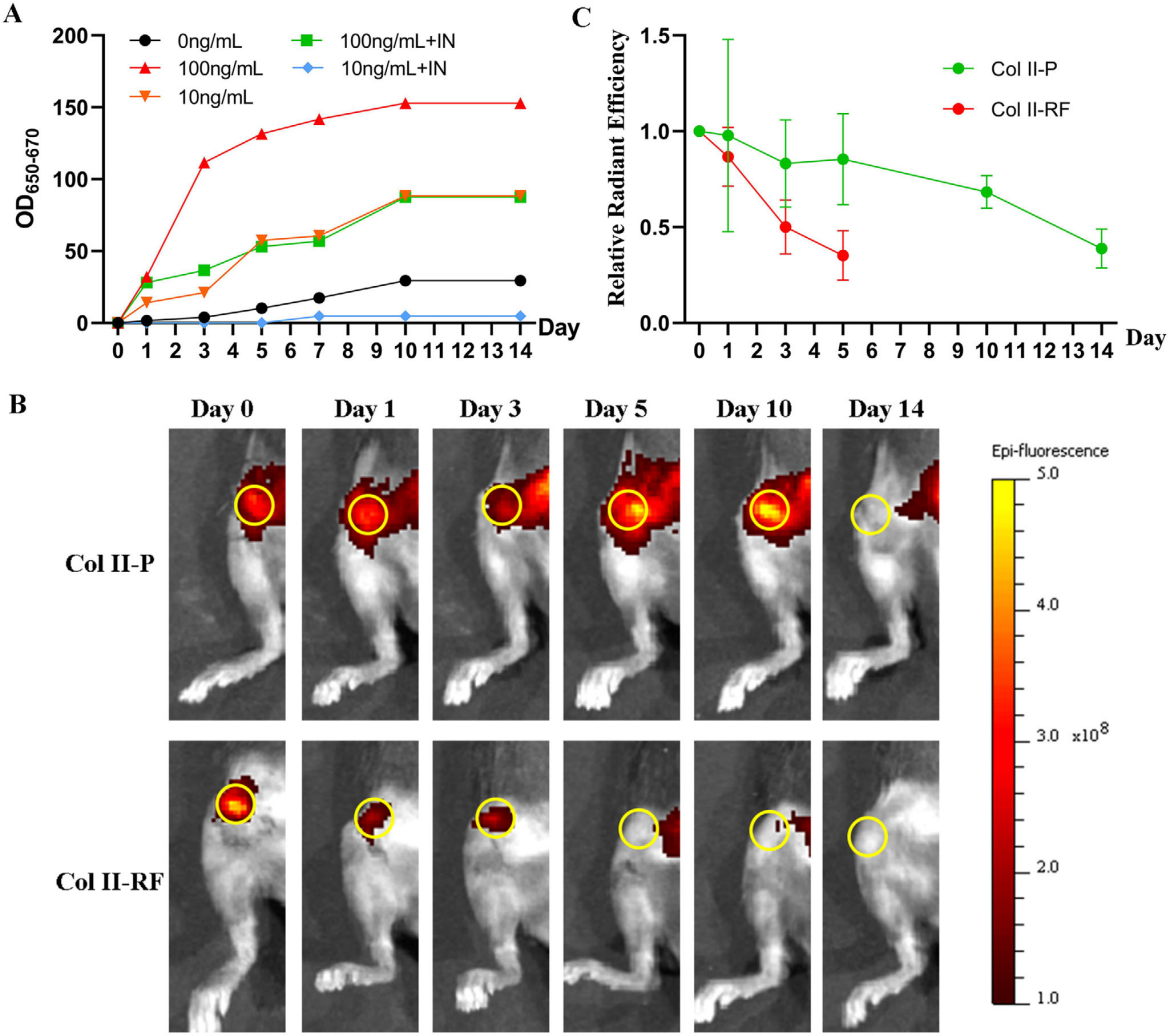
MMP13‐sensing drug release. (A) In vitro release kinetics of FGF‐18 from the scaffold in response to different concentrations of MMP13 and in the presence of an MMP13‐specific inhibitor. (B) In vivo MMP13‐responsive functionality of the scaffold in an OA cartilage defect model. (C) Relative fluorescence quantification analysis based on IVIS imaging (*n* = 3).

The MMP13‐sensing release capability and biocompatibility of the scaffold system were evaluated. As shown in Figure 2A, the in vitro release kinetics of FGF‐18, monitored by relative fluorescence intensity, demonstrate a clear dependence on MMP13 concentration. A rapid and substantial release of FGF‐18 is observed in the presence of a high concentration (100 ng mL^−1^) of MMP13. Crucially, this release is effectively abolished by the addition of an MMP13‐specific inhibitor (10 nm), confirming that the drug release is specifically mediated by MMP13 enzymatic activity and not by passive diffusion.

The MMP13‐responsive functionality was further validated in vivo (Figure 2B). RF and WYRGRL peptides with fluorescent tags were prepared. To demonstrate the MMP13‐responsivity of the Col II‐RF scaffold, a collagen type II scaffold bound with WYRGRL (Col II‐P) was used as a negative control. An OA model was established in mice eight weeks before scaffold implantation to induce inflammation and MMP13 expression in the joint. Subsequently, a cartilage defect was created in the mouse knee joint, and the scaffolds were implanted. The fluorescence intensity changes of the scaffolds were monitored using an in vivo small animal imaging system (IVIS) after implantation of both the Col II‐P and Col II‐RF scaffolds. As shown in the IVIS images, the Col II‐RF scaffold responds to MMP13 in the in vivo OA environment, releasing FGF‐18 into the joint cavity, which is then metabolized and cleared by day 5. In contrast, the Col II‐P scaffold, which does not respond to MMP13, shows a fluorescence intensity that declines slowly, reaching a level comparable to that of Col II‐RF only by day 14 (Figure 2C). As shown in Figure S3A, the cumulative release of FGF‑18 from the Col II‑RF scaffold, quantified by His‑tag ELISA, exhibits a clear MMP13 concentration‑dependent profile. This release is effectively abolished by the addition of the MMP13‑specific inhibitor, confirming that FGF‑18 liberation is specifically mediated by MMP13 enzymatic cleavage. For the dual‑loaded Col II‑RFC scaffold, FGF‑18 release also shows a concentration‑dependent, inhibitor‑sensitive trend. However, the release curve appears less smooth, likely owing to interference from the co‑loaded CXB (Figure S3B). Celecoxib concentration in the release medium was measured using a UV spectrophotometer. The data demonstrate an MMP13‑dependent trend (Figure S3C).

**FIGURE 3:**
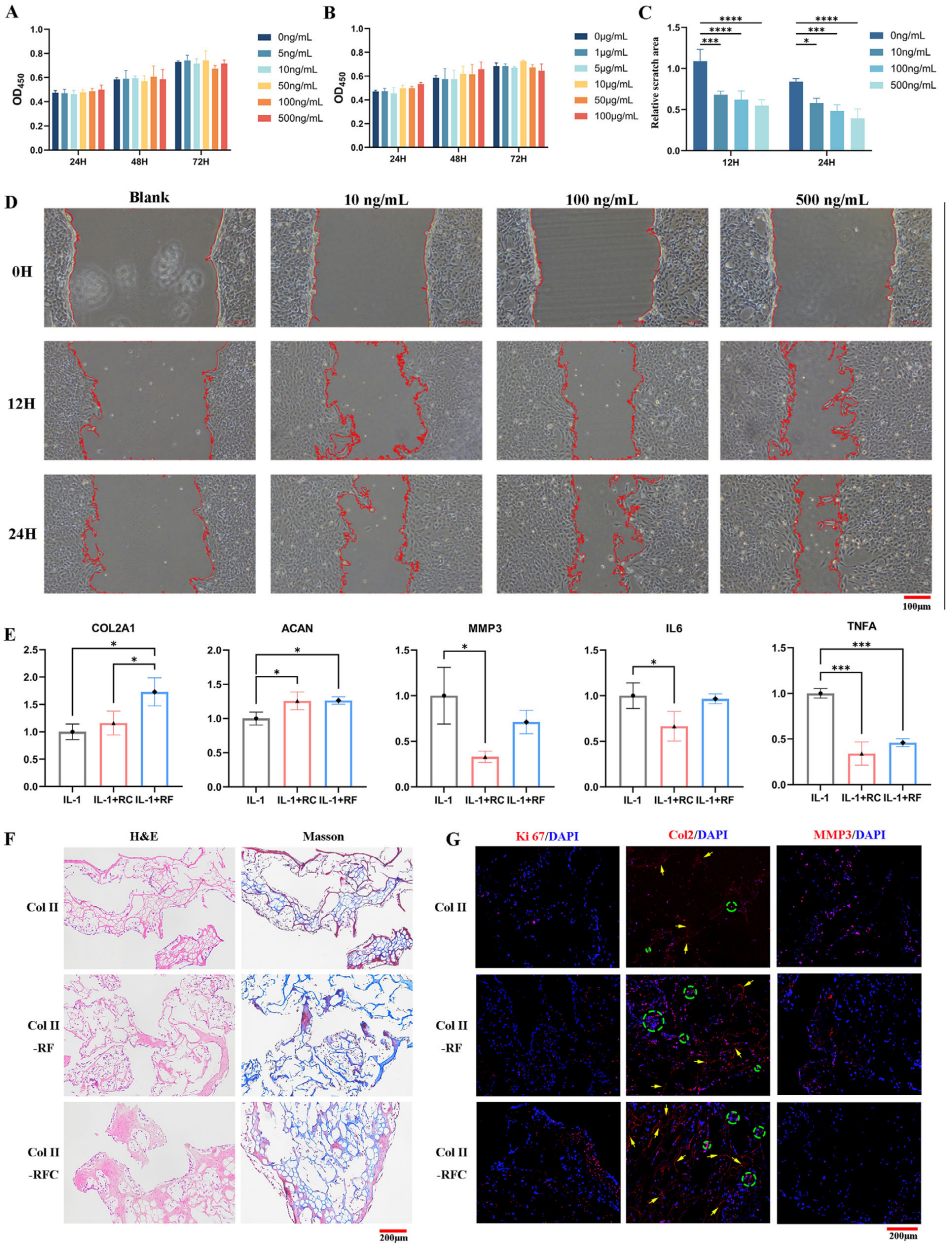
The bioactivity of the MMP13‐sensing scaffold system. The CCK‐8 assay (*n* = 3, two‐way ANOVA) showing the human chondrocyte viability after culture with different concentrations of RF (A) and RC (B) for 24, 48, and 72 h. (C) Quantitative analysis and (D) representative images of human chondrocyte migration in response to varying RF concentrations for 12 and 24 h (*n* = 3, one‐way ANOVA). (E) qPCR analysis of key anabolic and inflammatory genes of human chondrocytes stimulated with IL‐1β and treated with RC and RF (*n* = 3, one‐way ANOVA). (F) The H&E and Masson staining, and (G) immunofluorescence staining for Ki67, Col2, and MMP3 in human chondrocyte recellularized scaffolds under IL‐1β stimulation. Yellow arrows indicate the type II collagen scaffold; Green circles indicate type II collagen synthesized by chondrocytes. (*p < 0.05; **p < 0.01; ***p < 0.001; ****p < 0.0001).

Excellent biocompatibility is a prerequisite for any implantable scaffold. The cytotoxicity of the peptide‐drug conjugates, RF and RC, was assessed using the CCK‐8 assay on human chondrocytes. Cells were treated with a range of concentrations of RF (Figure 3A) and RC (Figure 3B) for 24, 48, and 72 h. The results show that cell viability remained consistently high across all tested concentrations and time points, indicating that neither conjugate elicited significant cytotoxic effects. These findings confirm the excellent in vitro biocompatibility of the designed drug delivery system.

### 2.3 In Vitro Anti‐Inflammatory and Pro‐Anabolic Effects

FGF‐18 significantly promotes chondrocyte migration, a critical process in early cartilage repair. The functional efficacy of the RF and RC was systematically evaluated through a series of in vitro bioactivity assays. This pro‐migratory effect of RF was visually confirmed by a scratch assay, which shows markedly accelerated closure of the wound gap in RF‐treated groups compared to controls after 12 and 24 h (Figure 3D). Statistical analysis indicates a dose‐dependent enhancement in human chondrocyte migration upon treatment with RF (Figure 3C). To evaluate the anti‐inflammatory and pro‐anabolic effects, human chondrocytes were stimulated with IL‐1β (10 ng mL^−1^) [20, 21] to model the osteoarthritic inflammatory microenvironment. qPCR analysis reveals that treatment with RC and RF effectively modulates gene expression (Figure 3E). RC significantly downregulates key inflammatory mediator genes (MMP3, IL6, TNFA) induced by IL‐1β, while concurrently upregulating the expression of essential anabolic genes, including COL2A1 and ACAN. RF significantly downregulates TNFA induced by IL‐1β, while also upregulating the expression of crucial anabolic genes, including COL2A1 and ACAN.

The therapeutic potential of the recellularized scaffolds was confirmed. H&E and Masson staining results indicate that chondrocytes adhered well to the scaffolds in all groups and were able to synthesize matrix (Figure 3F). Immunofluorescence staining for Ki67, type II collagen (Col2) and MMP3 in human chondrocytes cultured on the scaffolds under IL‐1β stimulation shows that cells on the functionalized scaffolds (Col II‐RF and Col II‐RFC) maintain significantly higher levels of Col2 synthesis compared to those on the Col II scaffold, highlighting the protective and pro‐chondrogenic role of the released drugs in an inflammatory environment (Figure 3G). The Col II‐RFC group exhibits the highest Ki67 fluorescence intensity, while the Col II group shows the lowest. Col2 staining reveals that the type II collagen scaffold in the Col II group is degraded, with the original scaffold structure no longer discernible. In contrast, the scaffold structure remains visible in the Col II‐RFC group. This indicates that RFC effectively delays the degradation of type II collagen under inflammatory and enzyme‐rich conditions. This finding is further supported by MMP3 immunofluorescence staining, which shows that the presence of RFC reduces MMP3 expression, leading to a decrease in enzymatic activity in the local microenvironment and consequently less type II collagen degradation. In contrast, in the Col II group, the presence of IL‐1β reduces chondrocyte proliferative activity (as indicated by low Ki67 expression) and increases MMP3 secretion, thereby accelerating type II collagen degradation.

**FIGURE 4:**
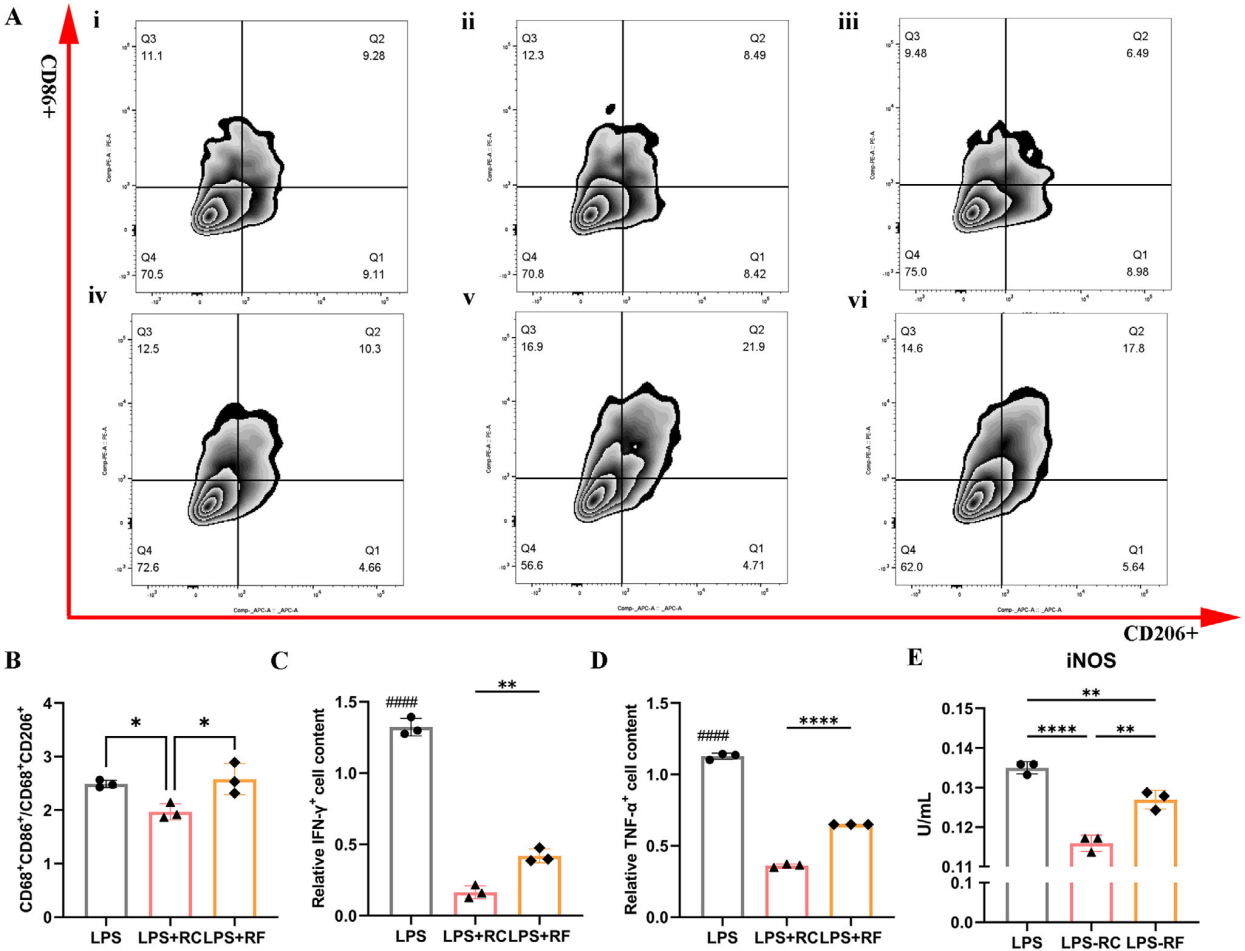
In vitro anti‐inflammatory efficacy of RC and RF. (A) Flow cytometry analysis to assess phenotypic changes in RAW264.7 cells without (i‐iii) or with (iv‐iv) LPS stimulation and subsequent treatment with RC (ii, v) and RF (iii, vi). (B) Quantitative analysis of the M1 macrophages (CD68^+^CD86^+^)/M2 macrophages (CD68^+^CD206^+^). Quantitative analysis of the IFN‐γ^+^ macrophages (C) and TNF‐α^+^macrophages(D). ELISA analysis of iNOS in macrophages. (*p < 0.05; **p < 0.01; ***p < 0.001; ****p < 0.0001, ^####^ *p* < 0.0001: LPS vs LPS+RC, and LPS vs LPS+RF groups. *n* = 3, one way ANOVA).

The immunomodulatory potential of the drugs was assessed on macrophages. Flow cytometry analysis of LPS‐stimulated RAW264.7 cells demonstrates the effects of RC (Figure 4Aii, v) and RF (Figure 4Aiii, vi) on macrophage polarization evaluated both in the absence (Figure 4Ai–iii) and presence (Figure 4Aiv–vi) of 100 ng mL^−1^ LPS [22]. In the absence of LPS, the ratio of CD68^+^CD86^+^/CD68^+^CD206^+^ cells in the RC group is significantly higher than that in the Blank and RF groups. In the presence of LPS, both the LPS+RC and LPS+RF groups exhibit similar ratios to the LPS‐only group (Figure S4). To assess the impact of RC and RF on Raw 264.7 cell polarization under LPS stimulation, the CD68^+^CD86^+^/CD68^+^CD206^+^ ratio in the absence of LPS was used as the baseline to calculate the relative changes after LPS addition. The results indicate that, compared to the LPS and LPS+RF groups, LPS+RC effectively lowers the CD68^+^CD86^+^/CD68^+^CD206^+^ ratio, reduces the proportions of IFN‐γ^+^ and TNF‐α^+^ macrophages, and decreases the level of iNOS in macrophages following LPS stimulation (Figure 4B–E), demonstrating its immunomodulatory effect.

### 2.4 In Vivo Therapeutic Efficacy in the OA Cartilage Defect Model

**FIGURE 5:**
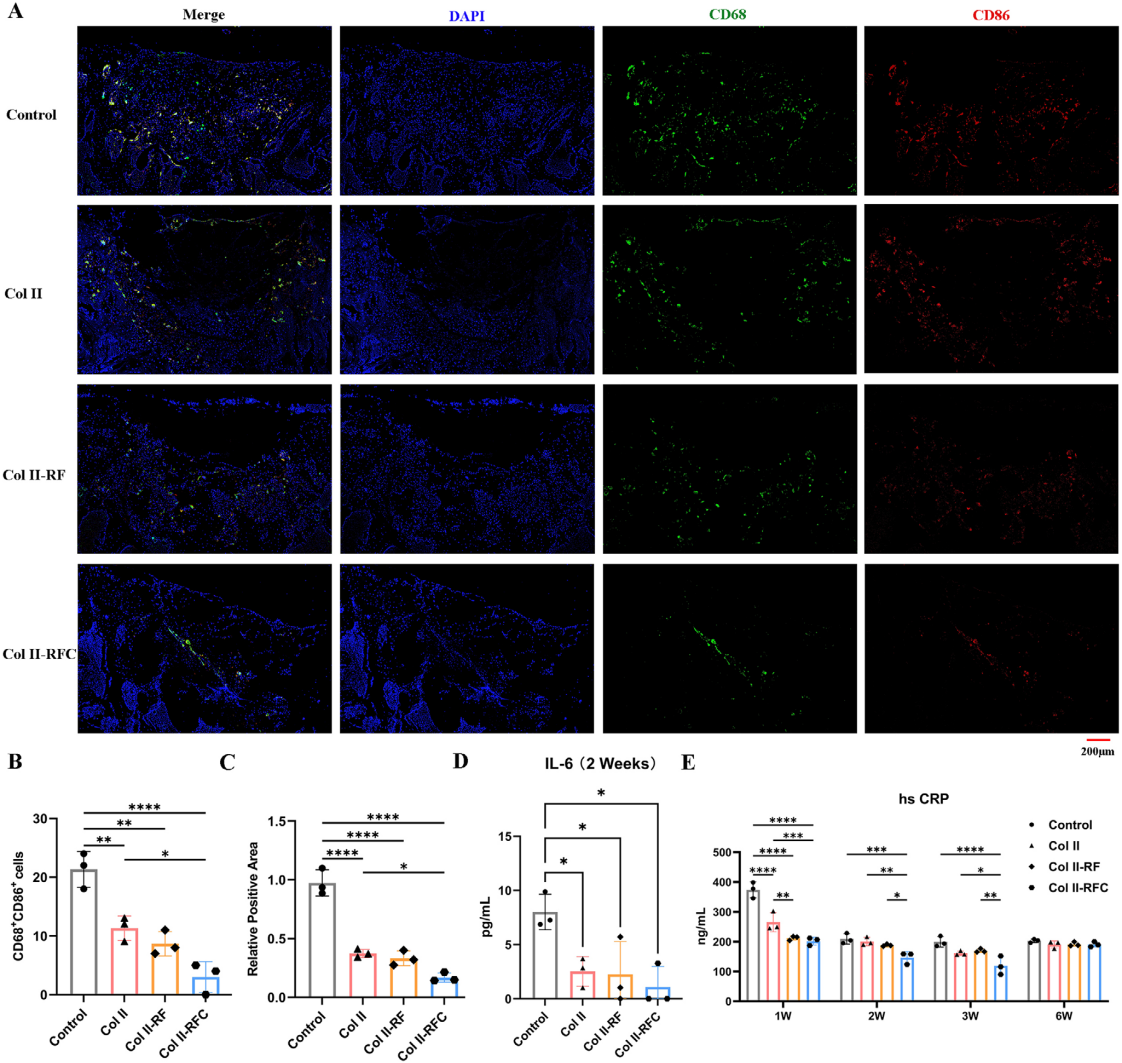
In vivo anti‐inflammatory efficacy of the MMP13‐sensing drug scaffold. (A) Immunofluorescence co‐localization staining of CD68 and CD86 at two weeks post‐ implantation. (B) Quantification of CD68^+^CD86^+^ cell numbers. (C) Quantification of the relative fluorescence area of CD68^+^CD86^+^ cells. ELISA analysis of IL‐6 (D) and hs‐CRP (E) in serum. (*p < 0.05; **p < 0.01; ***p < 0.001; ****p < 0.0001. *n* = 3, one ‐ way ANOVA and two‐way ANOVA).

The therapeutic efficacy of the MMP13‐sensing scaffold was rigorously evaluated in a rat model of OA cartilage defects. To investigate the immunomodulatory mechanism within the joint microenvironment, immunofluorescence co‐localization staining of macrophage markers was performed at two weeks post‐implantation (Figure 5A). The OA cartilage defects treated with the dual‐drug scaffold show markedly different macrophage numbers compared to controls (Figure 5B,C). This reduction in M1 macrophages underscores the scaffold's capacity to actively remodel the local immune milieu. Co‐localization analysis of CD68 and CD206 (Figure S5) reveals a virtual absence of CD68^+^CD206^+^ double‐positive cells within the defect area at 2 weeks post‐surgery. This finding indicates that M2‐like alternatively activated macrophages had not yet emerged in the local microenvironment at this early time point. Systemic inflammatory markers were assessed. As shown in Figure 5D,E, ELISA analysis of serum shows that animals treated with the dual‐drug scaffold (Col II‐RFC) exhibit significantly lower levels of the key inflammatory cytokines, interleukin‐6 (IL‐6) and high‐sensitivity C‐reactive protein (hs‐CRP), compared to the other groups. This indicates a potent anti‐inflammatory effect induced by the localized treatment.

**FIGURE 6:**
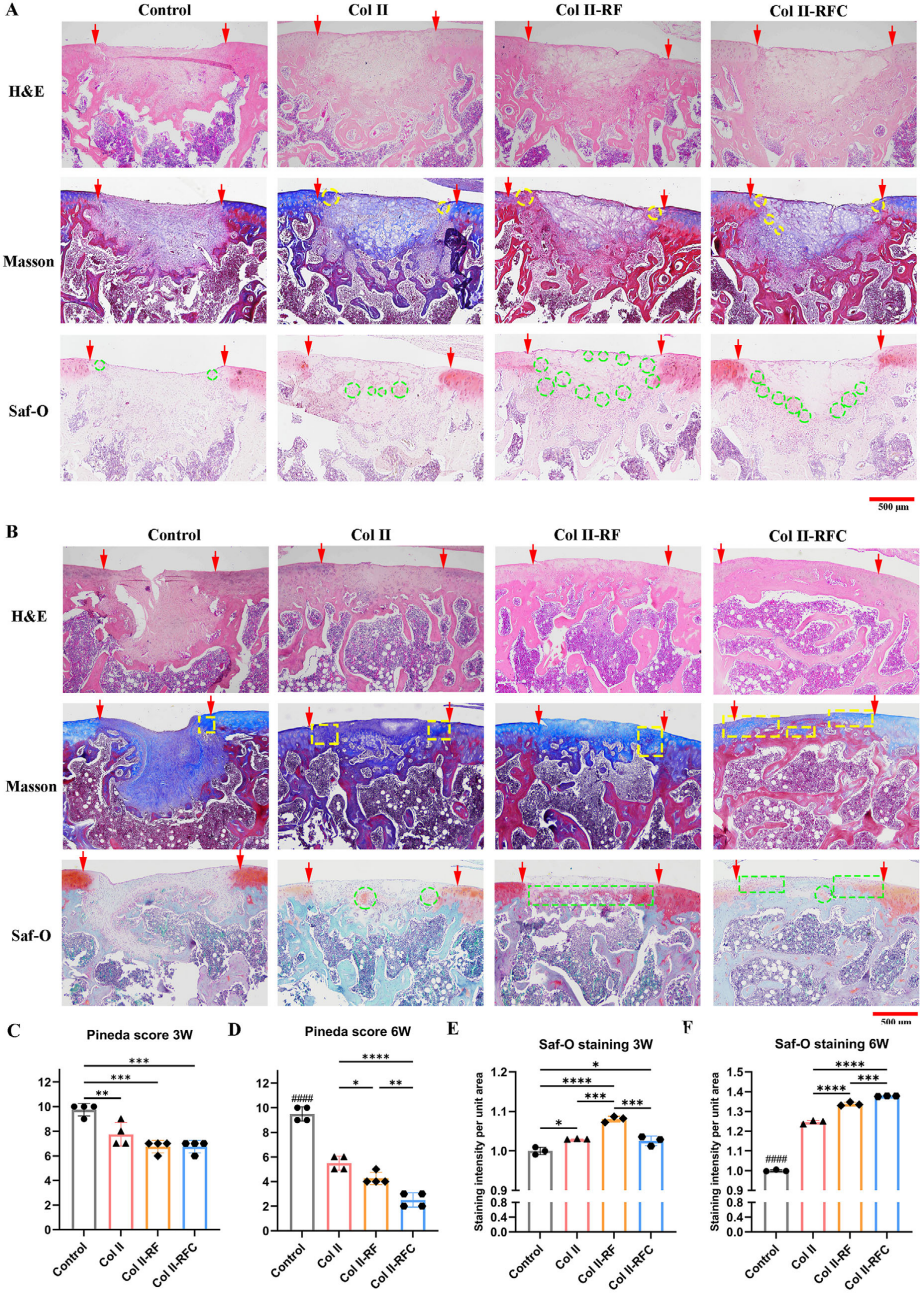
Histological evaluation of the repaired cartilage tissue. (A) H&E, Masson, and Saf‐O staining at three weeks and (B) six weeks after scaffold implantation. (Red arrows indicate the scaffold implantation site; Yellow boxes indicate collagen remodeling. Green boxes indicate GAG deposition). (C) Pineda scores at three weeks (*n* = 4), and (D) six weeks (*n* = 4) after implantation. (E) Saf‐O staining intensity analysis at three weeks (*n* = 3), and (F) six weeks (*n* = 3) after implantation. (*p < 0.05; **p < 0.01; ***p < 0.001; ****p < 0.0001; ^####^ *p* < 0.0001: Control vs Col II, Col II‐RF, and Col II‐RFC; one‐way ANOVA).

At three and six‐weeks post‐implantation (Figure 6), H&E staining of each group indicates that the cartilage defect sites in all groups were filled with matrix. Masson staining reveals that the defect area in the Control group is filled with irregularly oriented fibrous tissue. In contrast, the cartilage layers in the scaffold groups exhibit well‐structured fibers, resembling the surrounding native cartilage (yellow boxes show the sites with collagen remodeling). Safranin O staining and quantitative analysis (Figures 6E,F) show that more GAGs are deposited around the scaffolds in the Col II‐RFC and Col II‐RF groups, followed by the Col II group, while less GAG deposition is observed in the control group (Green boxes show the sites of deposited GAGs). Pineda scoring indicates comparable repair outcomes among all scaffold groups at three weeks post‐operation (Figure 6C). By six weeks, the Col II‐RFC group demonstrates the most favorable repair, followed by the Col II‐RF group, which performs better than the Col II group alone (Figure 6D).

**FIGURE 7:**
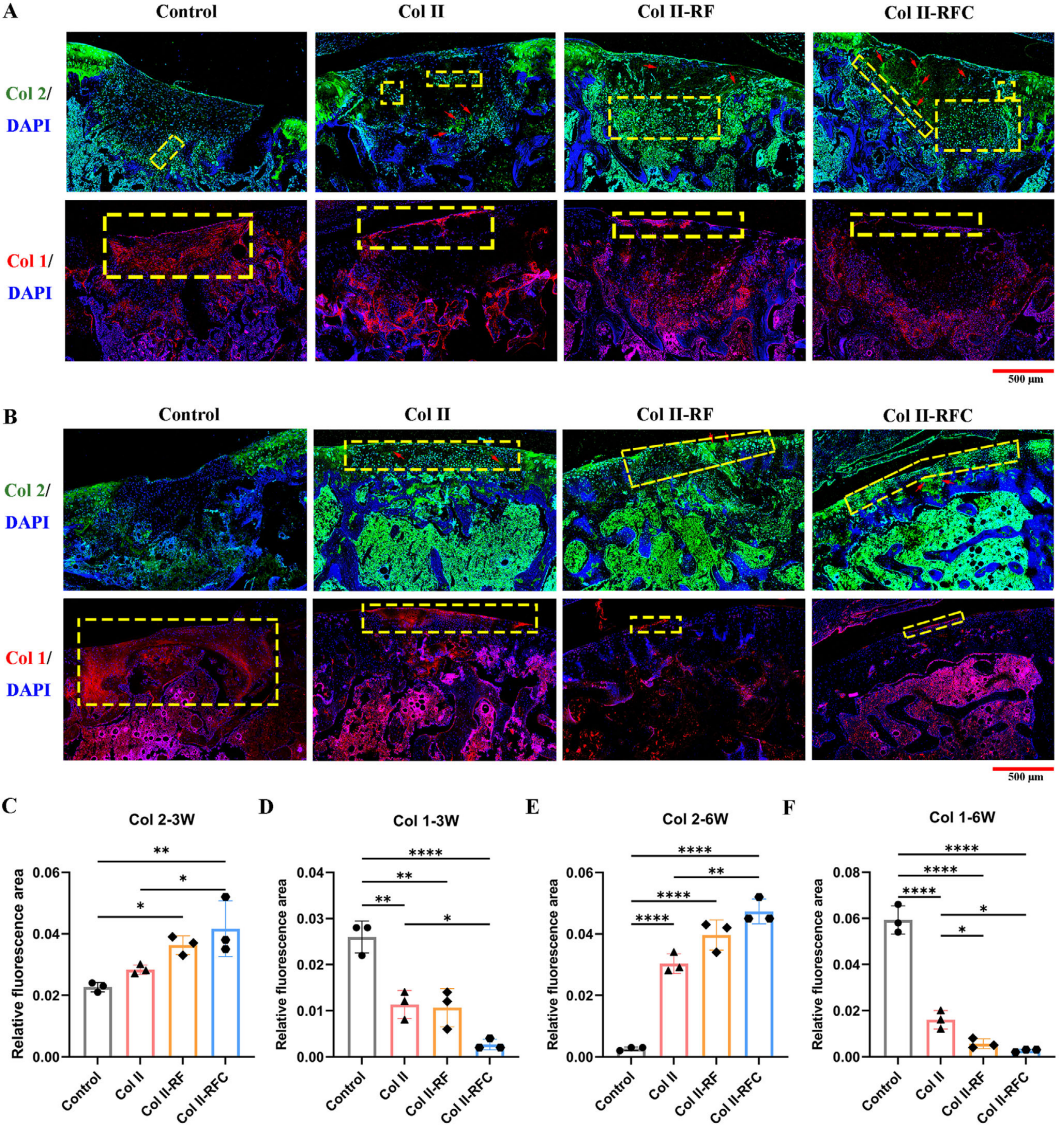
Immunofluorescence staining for type II collagen (Col2, green) and type I collagen (Col1, red) at three weeks (A) and six weeks (B) after scaffold implantation. Red arrows indicate the type II collagen scaffolds, and yellow boxes indicate the deposited collagen. Statistical analysis of the relative fluorescence area of ​​Col2 and Col1 at three weeks (C, D) and six weeks (E, F) post‐implantation. (*p < 0.05; **p < 0.01; ***p < 0.001; ****p < 0.0001. *n* = 3, one‐way ANOVA).

IF staining was utilized to characterize the collagen composition in the defect area, where green indicates type II collagen (Col2), red represents type I collagen (Col1), and blue labels cell nuclei (DAPI). IF results at three weeks post‐implantation (Figure 7A) reveal a mixed presence of both type I and type II collagen (Col1+Col2+) in the defect area of the control group. In contrast, the treatment groups show predominantly type II collagen (Col2+) at the three‐week point, while Col2 expression is slightly lower in the Col II group (Figure 7C). Notably, the tissue at the defect surface of the Col II and Col II‐RF groups exhibits a type I collagen phenotype (Col1+), whereas the Col II‐RFC group shows almost no Col1 signals (Col1+/‐) (Figure 7D).

By six weeks post‐implantation (Figure 7B), the defect site in the Control group exhibits strong type I collagen positivity and absence of type II collagen (Col1++Col2‐), while the Col II group displays a mixed collagen profile (Col1+Col2+). The Col II‐RF group demonstrates increased type II collagen deposition with type I collagen (Col1+Col2++). The Col II‐RFC group demonstrates increased type II collagen deposition, but less type I collagen deposition (Col1+/–Col2++) (Figure 7E,F).

**FIGURE 8:**
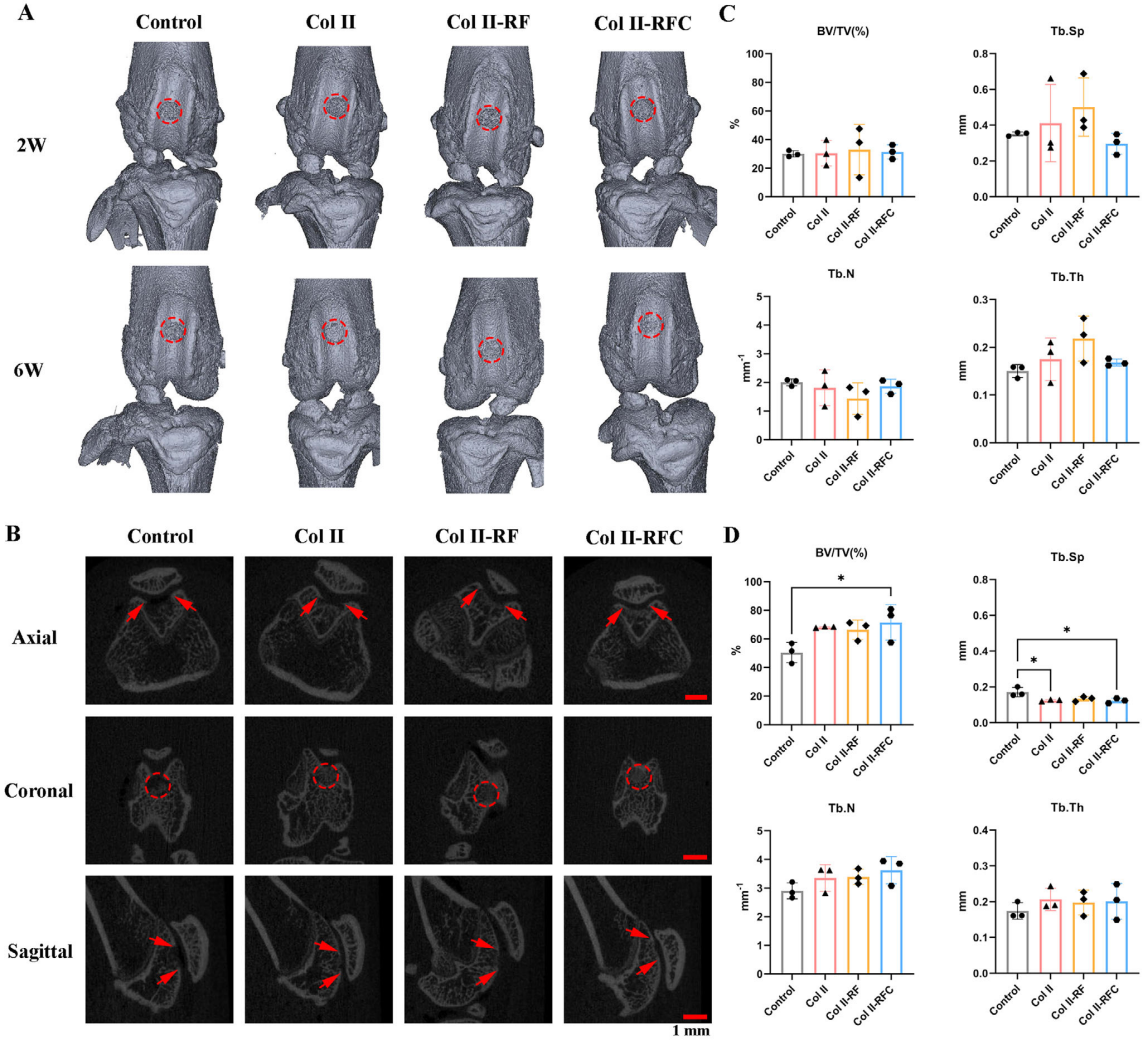
Micro‐CT analysis of rat knee joints at two‐, and six‐weeks post‐implantation. (A) Micro‐CT 3D reconstruction images of the knee joint. (B) Axial, coronal, and sagittal views of the subchondral bone at six weeks post‐implantation. (C) Quantitative analysis at two weeks, and six weeks (D) post‐implantation. Red arrows and red circles indicate the areas of interest. (*p < 0.05, *n* = 3, one‐way ANOVA).

The protective effect of the MMP13‐sensing dual‐drug scaffold on subchondral bone architecture was longitudinally monitored using micro‐CT over six weeks post‐implantation. Representative 3D reconstruction images of the rat knee joints are presented in Figure 8A, as well as 2D images in the axial, coronal, and sagittal planes for each group at six weeks post‐surgery (Figure 8B). Visually, the group implanted with the dual‐drug scaffold (Col II‐RFC) exhibits superior preservation of the subchondral bone structure and joint morphology at all time points compared to the control and single‐drug groups. The defect sites in the control groups show signs of progressive bone resorption and deterioration, whereas the dual‐drug scaffold group maintains a more intact and healthy‐appearing bony architecture. This qualitative observation is confirmed and quantified by analyzing the bone volume fraction (BV/TV), trabecular separation (Tb.Sp), trabecular thickness (Tb.Th), and trabecular number (Tb.N) [23, 24]. The quantitative data reveal a time‐dependent progression of bone healing; there are no statistically significant differences between the groups at two weeks post‐implantation (Figure 8C). The differences between the groups become apparent six weeks after surgery, and the BV/TV values in the dual‐drug scaffold group are significantly higher and Tb.Sp values are significantly lower than those in the OA control group (Figure 8D). These micro‐CT findings demonstrate that the dual‐drug scaffold facilitates cartilage repair and provides a crucial protective effect on the underlying subchondral bone, highlighting its comprehensive therapeutic potential in treating osteochondral defects.

### 2.5 Biocompatibility Evaluation of Scaffolds

**FIGURE 9:**
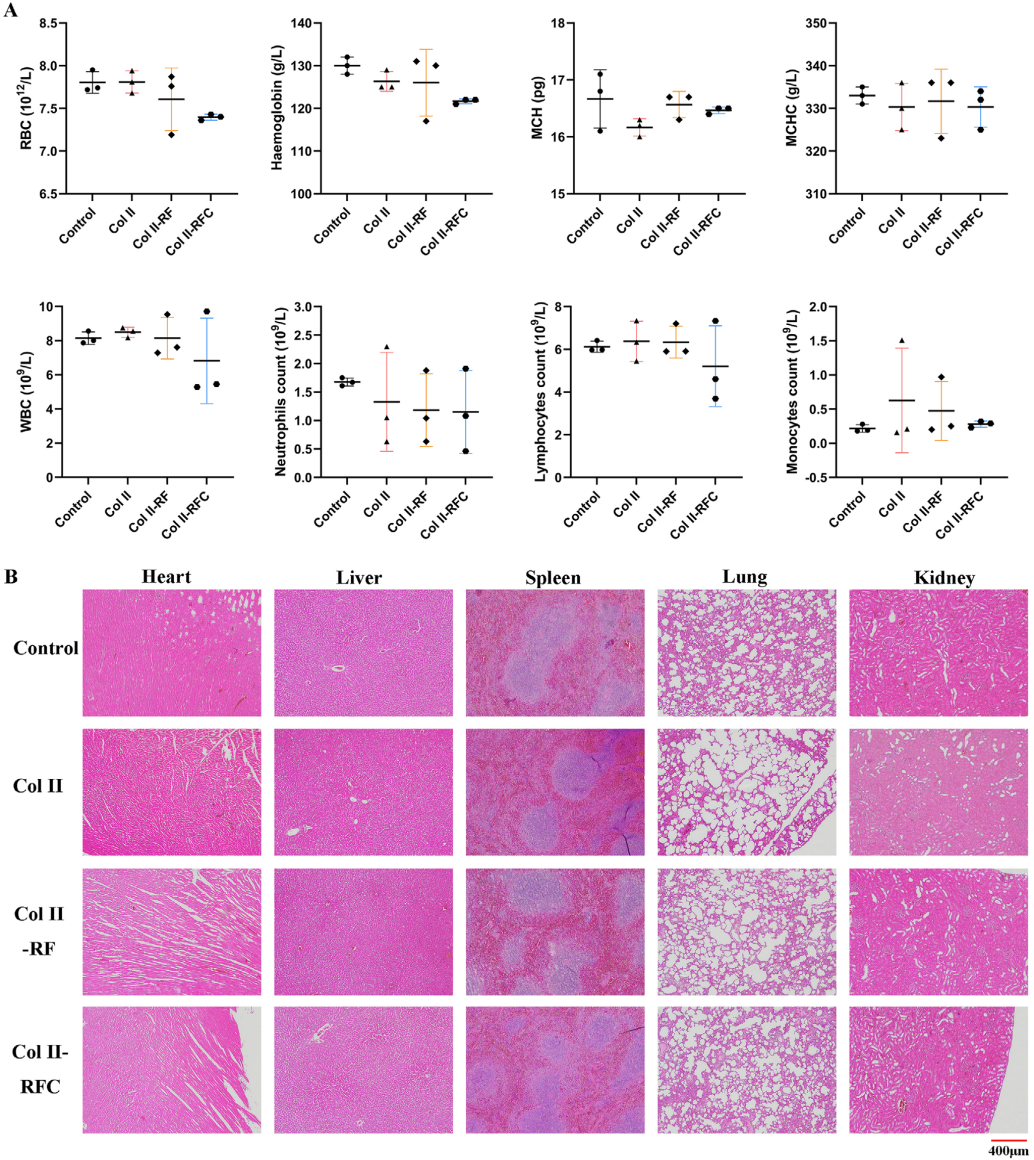
Long‐term biocompatibility of the scaffolds. (A) Complete blood count test (*n* = 3, one‐way ANOVA). (B) H&E staining of heart, liver, spleen, lung, and kidney tissues at 20 weeks post‐implantation.

The long‐term systemic biosafety of the implanted scaffolds was rigorously evaluated over 20 weeks. As shown in Figure 9A, a complete blood count (CBC) test was performed to assess potential hematological toxicity. All key parameters, including white blood cell (WBC) and red blood cell (RBC) counts, as well as hemoglobin, mean corpuscular volume (MCH), and mean hemoglobin concentration (MCHC) levels, fall within normal physiological ranges across all experimental groups. This result indicates the absence of systemic inflammatory reactions, anemia, or bone marrow toxicity induced by the scaffold or its degradation products. Furthermore, a comprehensive histological evaluation of major organs was conducted to confirm the lack of toxicity. Histopathological analysis via H&E staining reveals that the architecture of all examined organs, the heart, liver, spleen, lungs, and kidneys, remains normal and unremarkable (Figure 9B). These findings from hematological and histopathological analyses demonstrate the excellent long‐term biocompatibility and systemic safety profile of the MMP13‐sensing type II collagen scaffold, supporting its potential for future clinical translation.

## 3 Discussion

The present study demonstrates the successful development and comprehensive evaluation of a novel MMP13‐sensing, type II collagen‐based scaffold for the combinatorial treatment of osteoarthritic cartilage defects. The key innovation of this system lies in its sophisticated design that integrates biomimicry, pathology‐responsive drug release, and dual‐targeting therapy to disrupt the vicious cycle of OA and promote a pro‐repair microenvironment. The results collectively confirm that this intelligent scaffold effectively modulates the pathological OA environment by simultaneously combating inflammation and promoting anabolism, leading to significant functional repair of cartilage tissue.

Critically, the type II collagen scaffold serves as more than a drug carrier; it constitutes a native, biomimetic matrix essential for functional repair. Unlike synthetic polymers, its biochemical composition provides specific signals that promote chondrocyte adhesion and, crucially, maintain the chondrocytic phenotype, preventing dedifferentiation and facilitating the regeneration of hyaline‐like cartilage rather than fibrocartilage [25, 26, 27]. This intrinsic bioactivity synergizes with the released drugs to create a powerful pro‐regenerative microenvironment, highlighting the critical advantage of using a biologically active, tissue‐specific scaffold. The most significant finding is the MMP13‐specific, triggerable release of this system, which was rigorously validated both in vitro and in vivo. This mechanism ensures that therapeutic agents are released specifically at the site of disease activity, maximizing local bioavailability while minimizing systemic off‐target effects. This represents a major advance over conventional delivery systems, which often rely on passive diffusion and lack spatial‐temporal control. The subsequent synergistic efficacy of the two drugs proves to be crucial. CXB effectively suppresses the inflammatory cascade, as evidenced by reduced systemic and local levels of inflammatory markers. This suppression of catabolism creates a conducive environment for the anabolic actions of FGF‐18, which robustly stimulates chondrocyte migration, proliferation, and the synthesis of critical ECM components like type II collagen and GAG. The superior outcomes in the dual‐drug group, compared to either single‐agent group, underscore the necessity of this combinatorial approach to address the multifaceted pathology of OA. Furthermore, the scaffold demonstrates exceptional immunomodulatory capabilities, which are critical for long‐term repair success. The observed reduction of pro‐inflammatory M1 (CD86^+^) within the microenvironment indicates that the dual‐drug delivery scaffold actively remodels the immune landscape of the joint. This immunomodulatory effect is a key mechanism through which the scaffold mitigates disease progression and facilitates tissue healing. Furthermore, the type II collagen scaffold itself provides structural support for cell migration and proliferation. The quantitative comparison of repair outcomes clearly demonstrates the synergistic advantage of the Col II‐RFC. Pineda scoring reveals superior structural repair in the Col II‐RFC group at 6 weeks, outperforming Col II‐RF, Col II, and the control. Quantitative Safranin O staining unveils a dynamic, time‐dependent synergy: at 3 weeks, the Col II‐RF shows the highest GAG deposition due to FGF‐18 anabolic push, while by 6 weeks, the Col II‐RFC group achieves the most significant GAG accumulation. This shift indicates that CXB's sustained anti‐inflammatory action is crucial for preserving and maturing the newly synthesized matrix. Most compellingly, immunofluorescence quantification confirms that the Col II‐RFC group optimally promotes type II collagen synthesis while suppressing type I collagen deposition, yielding a collagen II/I ratio closest to native hyaline cartilage. These data converge to show that the sequential release of FGF‐18 (anabolism) and CXB (anti‐inflammation) creates a regenerative microenvironment that leads to qualitatively and quantitatively superior cartilage repair compared to single‐factor approaches. Beyond cartilage repair, the scaffold provides a protective effect on the subchondral bone. The significant improvement in BV/TV in the treatment group highlights the interconnectedness of cartilage and bone health in OA. By preventing the deterioration of the osteochondral unit, the scaffold ensures a more stable and functional foundation for the newly formed cartilage, addressing the pathology of OA more holistically. Finally, the excellent long‐term biocompatibility and systemic safety profile, confirmed by hematological analysis and histopathology of major organs, is a prerequisite for clinical translation and strongly supports the potential future application of this biomaterial.

Furthermore, the therapeutic outcomes achieved by the dual‐drug scaffold can be rationalized at the pathway level by the complementary mechanisms of its two payloads. Celecoxib, a selective COX‑2 inhibitor, suppresses the COX‑2/PGE2 signaling axis that is aberrantly activated in OA chondrocytes and synovial macrophages under inflammatory conditions. This inhibition reduces PGE2 production, a key pro‑inflammatory and catabolic mediator that drives the expression of matrix‑degrading enzymes [9, 28]. Concurrently, FGF‑18 binds with high affinity to FGFR3 on chondrocytes and activates the MEK/ERK1/2 signaling cascade, a pathway that has been shown to promote chondrocyte proliferation, induce the expression of cartilage‑specific matrix genes such as COL2A1 and ACAN, and suppress type I collagen expression, effects that collectively favor hyaline cartilage formation over fibrocartilage repair [29, 30, 31]. The convergence of these two distinct but complementary activities, inflammation quenching by CXB and matrix anabolism driven by FGF‑18. A key future perspective arising from this study pertains to the molecular mechanism of synergy between anti‐inflammatory suppression and anabolic stimulation mediated by FGF‐18 and CXB. The specific crosstalk and integrated signaling between their respective pathways remain to be fully elucidated. Future work will employ advanced in vitro pathomimetic models [32] or cartilage‐on‐a‐chip platforms [33] to dissect these synergistic mechanisms in depth.

The MMP13‐responsive type II collagen scaffold, engineered for the intelligent co‐delivery of celecoxib and FGF‐18, represents a paradigm shift in the therapeutic strategy for osteoarthritis. This system moves beyond symptomatic relief toward actual disease modification by leveraging a key pathological signal to trigger coordinated anti‐inflammatory and pro‐anabolic responses. The platform offers significant clinical advantages, including localized action that minimizes systemic exposure, a single implantation procedure, and compatibility with minimally invasive arthroscopic delivery. However, the clinical translation of this system would require addressing several essential steps. First, validation in large‐animal preclinical studies is crucial to assess functional repair in a model that more closely resembles human joint size and loading. Second, the peptide conjugation and drug‐loading process would need to be optimized for robustness and scalability to meet industrial‐scale manufacturing standards. Finally, further engineering may be necessary to ensure reliable performance in the complex human joint environment; for instance, extending the drug‐release window or incorporating additional microenvironmental triggers (e.g., pH) could enhance the system's responsiveness. Successfully addressing these points would position this responsive scaffold as a promising candidate for treating early‐stage osteoarthritic cartilage defects.

## 4 Conclusion

This study has successfully designed and fabricated an innovative MMP13‐sensing scaffold based on type II collagen for the on‐demand co‐delivery of celecoxib and FGF‐18. In a rat model of osteoarthritic cartilage defects, the scaffold demonstrated excellent MMP13‐triggered release, collectively combating inflammation and promoting anabolism to facilitate functional cartilage repair. This combinatorial strategy represents a paradigm shift from symptomatic management toward disease‐modifying therapy for osteoarthritic cartilage defects.

## 5 Materials and Methods

### 5.1 Fabrication of the MMP13‐Sensing Dual‐Drug Scaffold

#### 5.1.1 Synthesis and Characterization of Dual‐Functional Peptide (DFP)–Drug Conjugates

DFP: This peptide was designed by our team and custom‐synthesized by a commercial company (Sangon Biotech (Shanghai) Co., Ltd., China).

CXB conjugation (RC): The C‐terminal of the DFP‐COOH was conjugated to CXB (Y0001445) via a carboxyl‐amine coupling reaction using EDC (1‐ethyl‐3‐(3‐dimethylaminopropyl) carbodiimide) and NHS (N‐hydroxy succinimide) chemistry. Briefly, DFP was activated with EDC/NHS in PBS (pH = 5.5) for 15 min and then reacted with the CXB in DMSO for 2 h at room temperature. Here, each CXB molecule contains one ─NH_2_ group. To ensure efficient conjugation, CXB was used in a three‐fold molar excess over DFP, and EDC/NHS was employed in a five‐fold molar excess relative to the ─COOH groups on DFP. The conjugate (denoted as RC) was purified by acetone precipitation and characterized by ^1^H NMR in DMSO‐d6, which confirmed the presence of CXB aromatic protons (δ 7.2–7.8 ppm) and DFP backbone protons (δ 3.5–4.5 ppm).

FGF‐18 conjugation (RF): The DFP was conjugated to FGF‐18 (HY‐P7123) via a carboxyl‐amine coupling reaction using EDC and NHS chemistry. Briefly, FGF‐18 was activated with EDC/NHS in PBS (pH = 5.5) for 15 min and then reacted with DFP in PBS (pH = 7.4) for 2 h at room temperature. In the amino acid sequence of FGF‐18, there are 20 lysine residues bearing ─NH_2_ groups; among these, only the exposed ─NH_2_ groups are reactive. Assuming that each FGF‑18 molecule has 5 accessible ─NH_2_ groups available for conjugation, a 10‑fold molar excess of DFP relative to each available ─NH_2_ group was used to drive the reaction. EDC/NHS was employed in a 5‑fold molar excess relative to the ─COOH groups on DFP. The conjugate (denoted as RF) was purified using the desalting column (PD‐10) (Cytiva 17085101) to remove unreacted components. Successful synthesis was confirmed by pre‐attaching FITC fluorescence to the N‐terminus of DFP and Cy5 fluorescence (HY‐138200) to FGF‐18, followed by co‐localizing via fluorescence microscopy.

#### 5.1.2 Fabrication of Scaffolds

Col II scaffolds were prepared from a decellularized extracellular matrix derived from tissue‐ engineered hyaline cartilage grafts, following a previously described method [34]. Owing to the binding sequence WYRGRL in DFP, which binds to type II collagen, both RC and RF could be immobilized onto the type II collagen scaffold by co‐incubation. To obtain the Col II‑RF scaffold, RF was incubated with the Col II scaffold in PBS at 37°C for 12 h. Subsequently, to obtain the Col II‑RFC scaffold, RC was incubated with the Col II‑RF scaffold for an additional 12 h. Finally, the scaffolds were washed three times with PBS to remove any unbound RC and RF molecules.

### 5.2 Scaffold Characterization

#### 5.2.1 Scanning Electron Microscopy (SEM) Analysis

The morphology and surface topography of the scaffolds were examined using SEM. Scaffold samples were prepared by freeze‐drying to preserve the native porous architecture. Subsequently, the dried scaffolds were mounted on conductive adhesive tape and sputter‐coated with a thin gold layer (approximately 10 nm thick) to enhance surface conductivity and prevent charging effects. Imaging was performed at an accelerating voltage of 5 kV and a working distance of 8–10 mm.

#### 5.2.2 Fourier Transform Infrared (FTIR) and Raman Spectroscopy

Chemical functional groups and molecular interactions within the scaffolds were analyzed using FTIR spectroscopy. Spectra were acquired in attenuated total reflectance (ATR) mode over the range of 4000–500 cm^−^^1^. Molecular composition and structural integrity were further evaluated using Raman spectroscopy equipped with a 785 nm near‐infrared laser to minimize fluorescence background from biological samples. Spectra were collected in the range of 500–3500 cm^−^^1^.

#### 5.2.3 X‐Ray Photoelectron Spectroscopy (XPS)

XPS analysis was performed to determine the surface elemental composition and chemical states of the samples. Measurements were carried out on a Thermo Scientific K‐Alpha spectrometer (USA). Samples were cut into approximately 5 × 5 mm pieces and mounted on a sample holder. Analysis was initiated after the sample was transferred into the analysis chamber under ultra‐high vacuum (below 2.0 × 10^−^^7^ mbar). The X‐ray source was a monochromatic Al Kα beam with a spot size of 400 µm, operated at 12 kV and 6 mA.

#### 5.2.4 Quantification of CXB and FGF‑18 Conjugated to the Scaffold

A standard curve for celecoxib was established using a UV spectrophotometer [35, 36]. The RC solution before and after conjugation with the type II collagen scaffold was measured to assess the change in celecoxib content, thereby calculating the amount of celecoxib conjugated to the scaffold. For His‐tagged FGF‐18 (HY‐P73051), the His concentration was detected to derive the FGF‐18 content. First, the mass fraction of His in RF was determined. Then, the RF solution before and after conjugation with the type II collagen scaffold was analyzed using the His‐Tag ELISA kit (GenScript, L00436) to quantify the change in His content, from which the amount of FGF‐18 conjugated to the scaffold was calculated.

### 5.3 In Vitro and In Vivo Drug Release

#### 5.3.1 In Vitro Release Study

The drug release profile is a critical indicator of the scaffold's stimuli‐responsive functionality. The release kinetics of FGF‐18 from the Col II‐RF scaffold were investigated under conditions mimicking both the physiological (healthy) and pathological (OA) joint microenvironment. FGF‐18 was first labeled with Cy5. The release of FGF‐18 was quantified relatively by measuring the fluorescence intensity in the supernatant at different time points using a spectrophotometer (excitation 650 nm, emission 670 nm). Pathological OA joints are characterized by elevated MMPs, particularly MMP13, a key enzyme in cartilage collagen degradation. By incubating scaffolds in buffers containing 0, 10, or 100 ng mL^−1^ of MMP13, we aimed to simulate healthy (0 ng mL^−1^), mildly inflamed OA (10 ng mL^−1^), and severely diseased OA (100 ng mL^−1^) states. Crucially, an MMP13‐specific inhibitor (10 nm) was added to inactivate MMP13 in a parallel group. This design validates the scaffold's enzyme‐responsive, on‐demand drug release capability. The samples were incubated at 37°C under constant agitation (100 rpm) to maintain sink conditions and ensure homogeneity. The entire release medium was withdrawn and collected for analysis at predetermined time points (1, 3, 5, 10, and 14 d). The fluorescence intensity of the MMP13‐untreated group on the day 10 was set as 1, and the relative fluorescence intensity of each group at different time points was calculated to represent the FGF‐18 release capacity.

#### 5.3.2 In Vivo Release Study

The type II collagen‐blinding peptide WYRGRL and RF were conjugated to near‐infrared fluorescent dye (Cy5.5, HY‐D1389, and Cy5, HY‐138200, respectively). The fluorescently labeled peptides were then conjugated to the collagen type II scaffold. Two types of scaffolds were prepared: Col II‐P, Col II scaffold conjugated with WYRGRL, which lacked MMP13 sensitivity, served as the negative control group. Col II‐RF: Col II scaffold conjugated with RF, served as the test group. C57BL/6 mice were subjected to an established OA model to induce joint inflammation and upregulate endogenous MMP13 expression. Eight weeks post‐OA modeling, when OA pathology and MMP13 expression were confirmed, a second surgery was performed to create a full‐thickness cartilage defect (0.5 mm in diameter) in the femoral trochlea of the OA knee. The pre‐prepared scaffolds (Col II‐RF or Col II‐P) were then implanted press‐fit into the defect site. Sham‐operated animals (without OA induction or scaffold implantation) served as additional controls for background fluorescence. The in vivo release of the fluorescent tag (as a surrogate for the conjugated drug, FGF‐18) was non‐invasively monitored using an IVIS Spectrum system. Imaging was performed at predetermined time points post‐implantation: 0,1, 3, 5, 10, and 14 d. Before each imaging session, mice were anesthetized with isoflurane. A standardized region of interest (ROI) was drawn around the implanted knee joint, and the total radiant efficiency ([p/s]/[µW/cm^2^]) was quantified using IVIS Living Image software.

### 5.4 In Vitro Biological Evaluation

#### 5.4.1 Cell Culture

The human chondrocyte cell line C20A4 for anabolism promotion and migration study was cultured in DMEM supplemented with 10% fetal bovine serum (FBS) and 1% penicillin‐streptomycin. Cells between passages 3–5 were used to maintain phenotypic stability. The RAW264.7 cell line for the immunomodulation study was culturedin DMEM containing 10% FBS and 1% penicillin‐streptomycin.

#### 5.4.2 Biocompatibility Assessment

Cell viability was quantitatively assessed using the CCK‐8 assay, which measures mitochondrial activity via water‐soluble formazan production. Cells were seeded in 96‐well plates at a density of 4000 cells per well and incubated for 24, 48, and 72 h with different concentrations of RC and RF, followed by CCK‐8 assay.

#### 5.4.3 Cell Migration Assay

Cell migration capability, critical for cell recruitment and defect integration, was evaluated using a scratch assay. Chondrocytes were grown to confluence in six‐well plates, and a standardized scratch was created using a sterile pipette tip. Cells were then treated with different concentrations of RF. Wound closure was monitored at 0, 12, and 24‐ h using phase‐contrast microscopy, and the migration rate was quantified by measuring the reduction in scratch area using ImageJ software.

#### 5.4.4 Anti‐Inflammatory Assay

To simulate the osteoarthritic inflammatory microenvironment, chondrocytes were pre‐stimulated with 10 ng mL^−1^ IL‐1β for 48 h to induce catabolic and inflammatory responses. Cells were then treated with either 500 ng mL^−1^ RF or 100 µg mL^−1^ RC for an additional 24 h. The expression of key inflammatory mediator genes (MMP3, IL‑6, and TNF‑α) was quantified by qPCR (Table S1).

The impact of RC and RF on RAW 264.7 cell polarization was evaluated under both non‐polarized and M1‐polarized conditions, the latter induced by 100 ng mL^−1^ LPS. In the polarization group, cells were treated with 100 ng mL^−1^ LPS together with either 500 ng mL^−1^ RF or 100 µg mL^−1^ RC for 48 h. Subsequently, the proportions of CD68^+^CD86^+^ and CD68^+^CD206^+^ cells were analyzed by flow cytometry. The following antibodies were used: CD68 (MA5‐16676), CD86 (12‐0862‐82), and CD206 (17‐4031‐82). Pro‐inflammatory markers TNF‑α (11‑7349‑81) and IFN‑γ (12‑7319‑41) were detected by flow cytometry, and iNOS (SEKM‑0270) was detected by ELISA.

#### 5.4.5 Anabolic Activity

To simulate the osteoarthritic inflammatory microenvironment, chondrocytes were pre‐stimulated with 10 ng mL^−1^ IL‐1β for 48 h. Cells were then treated with either 500 ng mL^−1^ RF or 100 µg mL^−1^ RC for an additional 24 h. The expression of key anabolic genes (COL2A1, ACAN) was quantified by qPCR (Table S1).

For 3D culture, chondrocytes (10^6^ cells in 10 µL) were seeded onto scaffolds placed in 12‐well plates and allowed to adhere during a 5 h incubation. Subsequently, 2 mL of medium containing 10 ng mL^−1^ IL‐1β was added to each well. To comprehensively evaluate the scaffolds' anabolic and anti‐inflammatory properties, the cell‐scaffold constructs were cultured for 3 days and then analyzed by IF staining for Ki67 (ab15580), type II collagen (Col2, ab34712), and MMP3 (Proteintech, 17873).

### 5.5 In Vivo Animal Study

#### 5.5.1 Animal Modeling

The City University of Hong Kong Animal Care and Use Committee approved all animal procedures. Eight‐week‐old male Sprague‐Dawley rats were selected for this study due to their established utility in OA research, particularly in surgical induction models replicating human OA pathophysiology. Osteoarthritis was induced in the knee via anterior cruciate ligament transection (ACLT). This model effectively causes joint instability and progressive cartilage degeneration, mimicking the mechanical and inflammatory aspects of human OA. Eight weeks after ACLT (successful OA modeling confirmed), a second surgery was performed to create a cartilage defect (diameter = 1.5 mm, depth = 0.5 mm, critical size) in the femoral trochlea, The animals were randomly divided into four groups: (1) OA Control group: ACLT +cartilage defect without treatment; (2) OA +cartilage defect+ Col II scaffold: Implanted with a type II collagen scaffold. (3) OA +Cartilage defect+ Col II‐RF: Scaffold loaded with FGF‐18. (4) OA +Cartilage defect+ Col II‐RFC: Scaffold dual‐loaded with FGF‐18 and CXB.

#### 5.5.2 Serum Collection for ELISA

Blood samples were collected from the abdominal aorta under anesthesia at 1‐, 2‐, 3‐, and 6‐weeks post‐scaffolds implantation. After coagulation and centrifugation, serum was obtained. Inflammatory biomarkers including high‐sensitivity C‐reactive protein (hs‐CRP) and interleukin‐6 (IL‐6) were quantified using ELISA kit (Fine Test ER1048 and EH0201).

#### 5.5.3 Histological and Immunofluorescence Analyses

Rat knee joint samples were collected 2, 3, and 6 weeks after scaffolds implantation. The animals were euthanized with carbon dioxide, and the samples were subjected to routine fixation, decalcification, and paraffin embedding. Tissue sections were stained with H&E, Safranin O, and Masson's trichrome to evaluate tissue integrity, matrix composition, and fibrosis. Inflammation and macrophage polarization were analyzed by immunofluorescence using antibodies against CD68 (MA5‑16676), CD86 (12‑0862‑82), and CD206 (17‑4031‑82). The collagen phenotype in the cartilage defect area was characterized by immunofluorescence using antibodies against Col1 (ab270993) and Col2 (ab34712). The secondary antibody was Goat Anti‑Rabbit IgG H&L (ab150079).

#### 5.5.4 Micro‐CT Scanning

Knee joints were collected 2‐, and 6‐weeks post‐implantation and scanned using the Scanco Xtreme II micro‐CT scanner system. Subchondral bone microstructure was analyzed using CTAn software for bone volume fraction (BV/TV) and trabecular separation (Tb.Sp), trabecular thickness (Tb.Th), trabecular number (Tb.N). 3D reconstruction was performed using Mimics software.

#### 5.5.5 Long‐Term Biocompatibility

To assess the long‐term biocompatibility and systemic biosafety of the implanted scaffolds, the animals were maintained for 20 weeks post‐implantation. Before euthanasia, whole blood was collected from the abdominal aorta under deep anesthesia for a complete blood count (CBC). Following euthanasia, major organs (heart, liver, spleen, lungs, and kidneys) were carefully harvested, rinsed in saline, and fixed in 4% paraformaldehyde. After fixation, the organs were paraffin‐embedded, sectioned, and stained with H&E.

### 5.6 Statistical Analysis

All quantitative data are presented as mean ± standard deviation (SD). Statistical significance was determined using one‐way or two‐way ANOVA followed by Tukey's post‐hoc test for multiple comparisons. All tests were two‐sided, with a significance level α = 0.05. All analyses were performed using GraphPad Prism 9.0. A statistically significant difference was considered at a minimal level of significance of *p* < 0.05, and denoted as * *p* < 0.05, **p < 0.01, *** *p* < 0.001, **** *p* < 0.0001.

## Author Contributions

**Zhen Zhang**: writing – original draft preparation, writing – review & editing, formal analysis, methodology, software, investigation, and data curation. **Bangheng Liu**: writing – review & editing, animal experimentation, investigation, and data curation. **Yulei Mu**: validation methodology. **Huiqun Zhou**: validation and methodology. **Liang Ma**: methodology. **Chenjie Xu**: resources, writing – review & editing. **Dong‐An Wang**: resources, writing – review & editing, funding acquisition, supervision, project administration, and conceptualization.

## Funding

This work was supported by General Research Fund (GRF 11205324), Research Grants Council (RGC) / University Grants Council (UGC), Hong Kong SAR; Grant from Karolinska Institutet Ming Wai Lau Centre of Reparative Medicine (CityUHK 9231412); Grant from Health@InnoHK: CNRM, Innovation and Technology Commission, Hong Kong SAR.

## Ethics Statement

All animals used in this study were provided by the Laboratory Animal Research Unit (LARU) and were conducted following the regulations of the Department of Health. All animal experiments have been approved by the Animal Research Ethics Committee of the City University of Hong Kong (Approval No: AN‐STA‐00000025).

## Conflicts of Interest

The authors declare no conflicts of interest.

## Supporting information




**Supporting File**: advs76406‐sup‐0001‐SuppMat.doc.

## Data Availability

The data that support the findings of this study are available from the corresponding author upon reasonable request.
