# Supplementary material for: Combating Inflammation and Promoting Anabolism in Osteoarthritic Cartilage Defect With an MMP13‐Sensing Dual‐Drug Scaffold
Source: Adv Sci (Weinh). 2026 Jul 13:e19950. Online ahead of print. doi: 10.1002/advs.202519950 (PMC13364735; doi:10.1002/advs.202519950)
Supplement: Supplementary file 1 — Supporting File: advs76406‐sup‐0001‐SuppMat.doc. [file ADVS-9999-e19950-s001.doc]

| Gene | Primer sequence (both 5′-3′) |
| --- | --- |
| Human *COL2A1* | F: CTATCTGGACGAAGCAGCTGGCA  R: ATGGGTGCAATGTCAATGATGG |
| Human *ACAN* | F: GGTACCAGTGCACAGAGGGGTT  R: TGCAGGTGATCTGAGGCTCCT |
| Human *MMP3* | F:CAAAACATATTTCTTTGTAGAGGACAA  R: TTCAGCTATTTGCTTGGGAA |
| Human *IL6* | F: GGAGACTTGCCTGGTGAAA  R: CTGGCTTGTTCCTCACTACTC |
| Human *TNFA* | F: CCCATGTTGTAGCAAACCCTC  R: TATCTCTCAGCTCCACGCCA |
| Human *GAPDH* | F: AAGGTGAAGGTCGGAGTCAAC  R: GGGGTCATTGATGGCAACAATA |

**Table S1** qRT-PCR primer sequences: Forward (F) and Reverse (R)


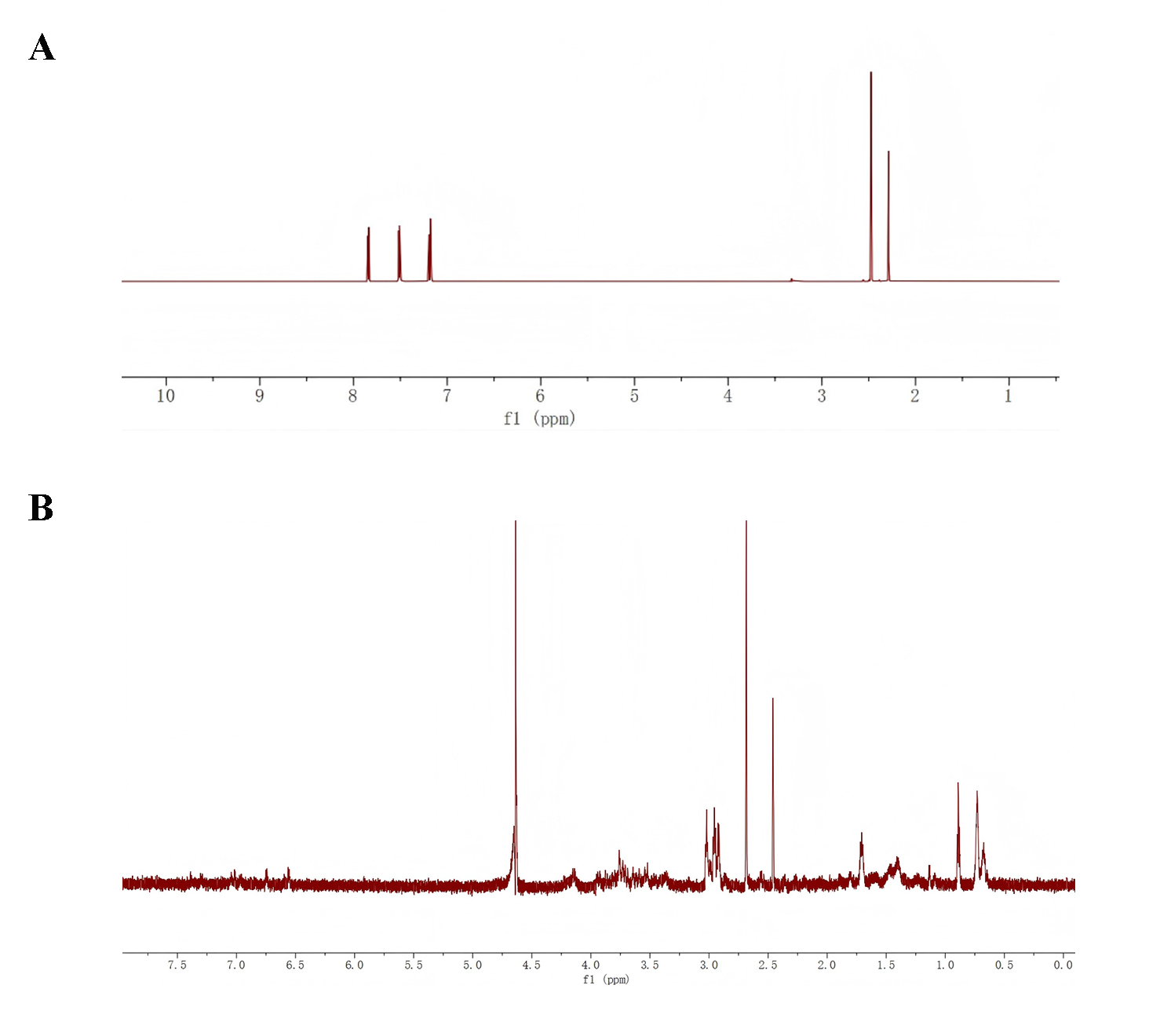


**Figure S1** The 1H NMR analysis of CXB (A) and RF (B).


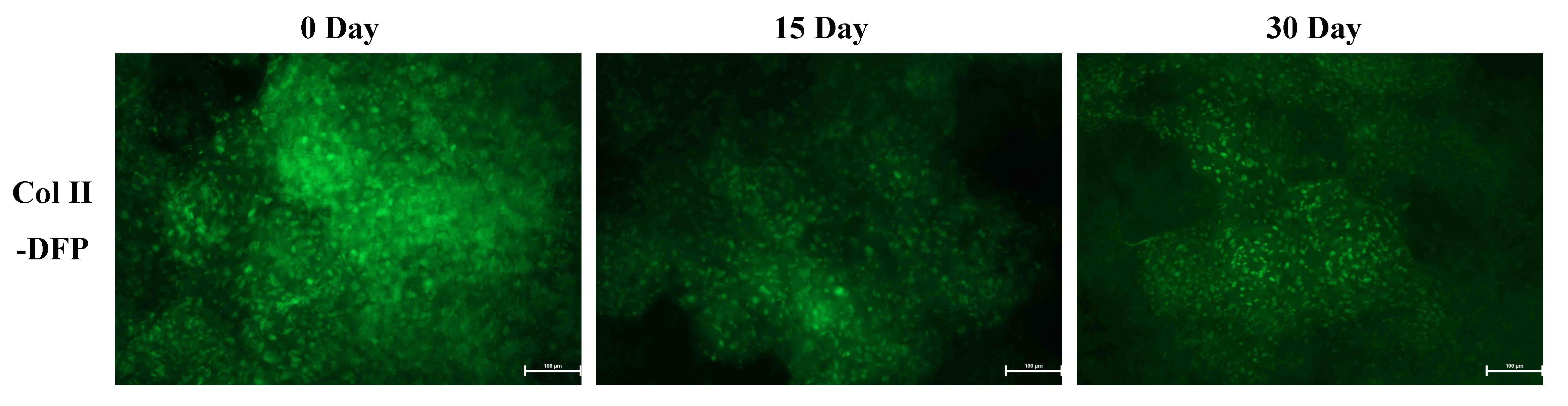


**Figure S2** The IF assay evaluates the specific binding ability of DFP (labeled with FITC, green) to type II collagen scaffolds.


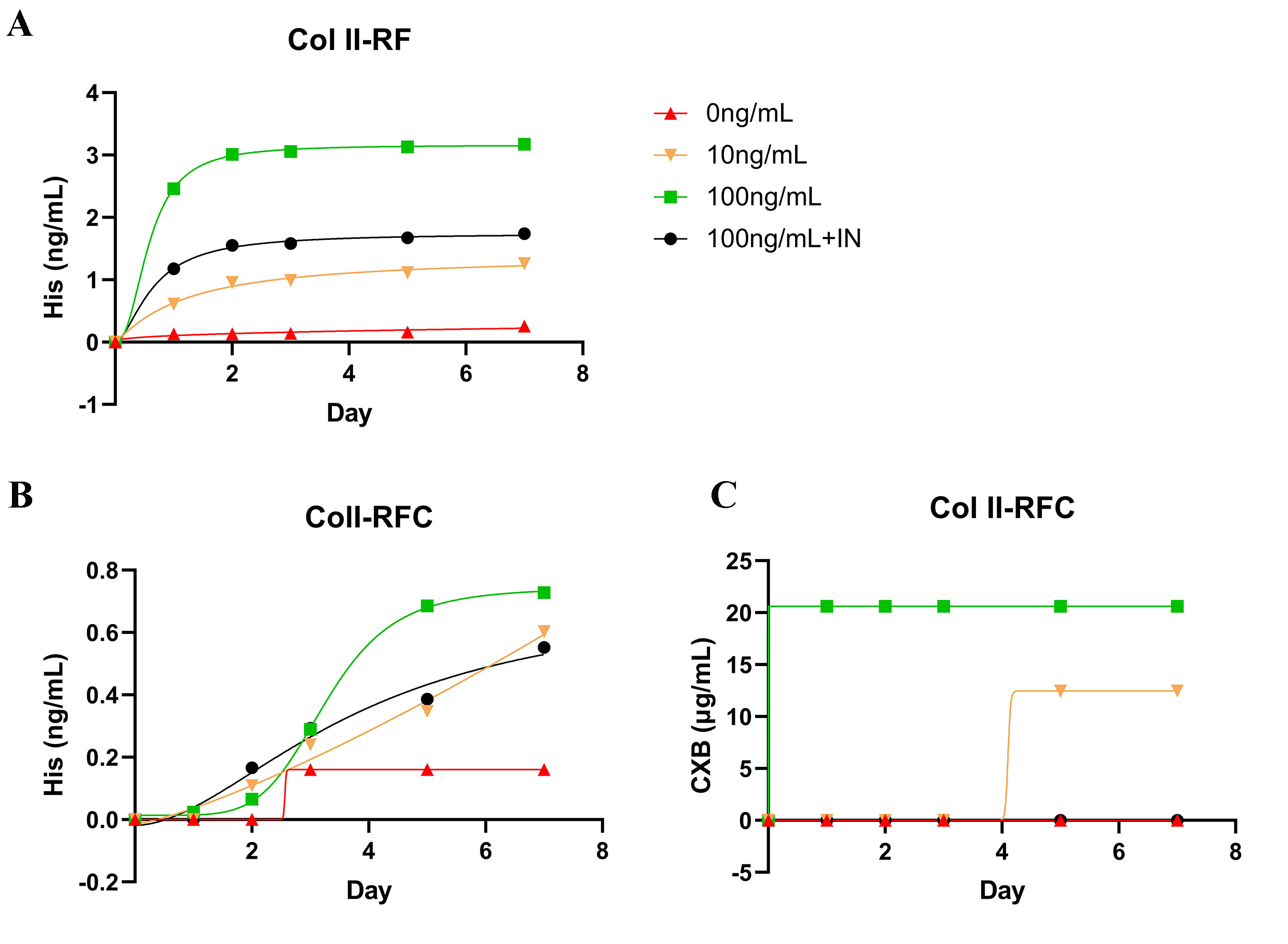


**Figure S3** MMP13-sensing cumulative release curves of Col II-RF(A) and Col II-RFC(B and C).


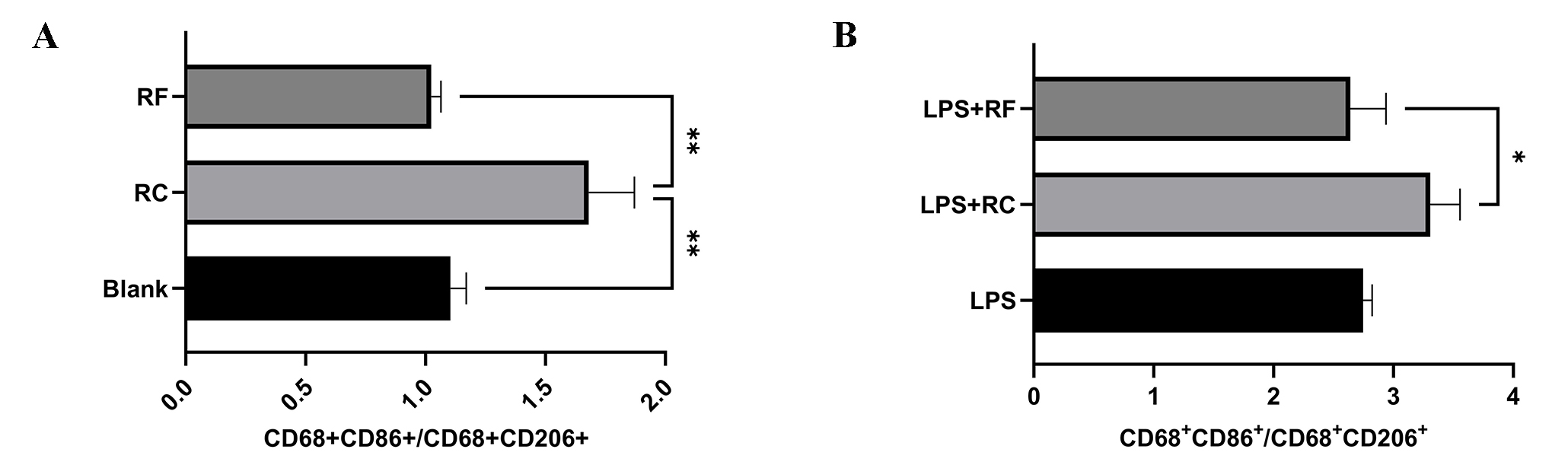


**Figure S4** The quantitative analysis of the M1 macrophages (CD68+CD86⁺)/M2 macrophages (CD68⁺CD206⁺) without LPS (A) and with LPS (B).

**Figure S5** Immunofluorescence co-localization staining of CD68 and CD206 two weeks after scaffolds implantation.
